# Facial Paralysis

**Published:** 1892-11

**Authors:** 


### Facial Paralysis.

Dr. Lusanna (*Rivista Veneta*) makes quite an extended study of the course and disease of the seventh pair of cranial nerves. He divides it anatomically into five portions:

1. Cerebral.
2. Bulbar.
3. Intra-cranial; the trunk of the nerve from its root to its entrance into the internal auditory meatus.
4. Inter-cranial; its traverse through the cranium.
5. Extra-cranial, from the stylo-mastoid foramen to its different terminal filaments.

These divisions are of much importance, not only from a diagnostic point of view, but also in rendering a prognosis. Lesions occurring in the second portion are often fatal; those in the first, third and fourth are serious, while those in the fifth are light.

The differential symptoms indicating the seat of the disease in its course, are:

1st Portion.—Cerebral, conservation of reflex phenomena.

2d Portion.—Bulbar, paralysis of the extremities.

3d Portion.—Intra-cranial, injury to the neighboring nerves.

4th Portion.—Inter-cranial, gustation of the anterior part of the tongue abolished. Hyperesthesia of audition.

5th Portion.—Extra-cranial, paralysis of the facial muscles not of the palate.

